# [Corrigendum] AHIF promotes glioblastoma progression and radioresistance via exosomes

**DOI:** 10.3892/ijo.2026.5920

**Published:** 2026-07-15

**Authors:** Xuejun Dai, Keman Liao, Zhijun Zhuang, Binghong Chen, Zhiyi Zhou, Sunhai Zhou, Guoshi Lin, Feifei Zhang, Yingying Lin, Yifeng Miao, Zhiqiang Li, Renhua Huang, Yongming Qiu, Ruisheng Lin

Int J Oncol 54: 261-270, 2019; DOI: 10.3892/ijo.2018.4621

Following the publication of the above paper, it was drawn to the Editor's attention by a concerned reader that, in Fig. 2E on p. 264, the Transwell invasion assay results shown in the 'T98G-AHIF KD' data panel appeared to potentially contain an overlapping section with the 'U251-NC' data panel in Fig. 3E.

After having re-examined their original data, the authors have realized that Fig. 3 of the above paper was inadvertently assembled incorrectly. The revised version of Fig. 3, now showing replacement data for Fig. 3E (the Transwell results for the U251-NC and U251-AHIF-OE experiments), is featured on the next page. Note that the error made in assembling Fig. 3 did not adversely affect either the results or the overall conclusions reported in this study. All the authors agree with the publication of this corrigendum, and are grateful to the Editor of *International Journal of Oncology* for allowing them the opportunity to publish this. They also wish to apologize to the readership of the Journal for any inconvenience caused.

## Figures and Tables

**Figure 3:**
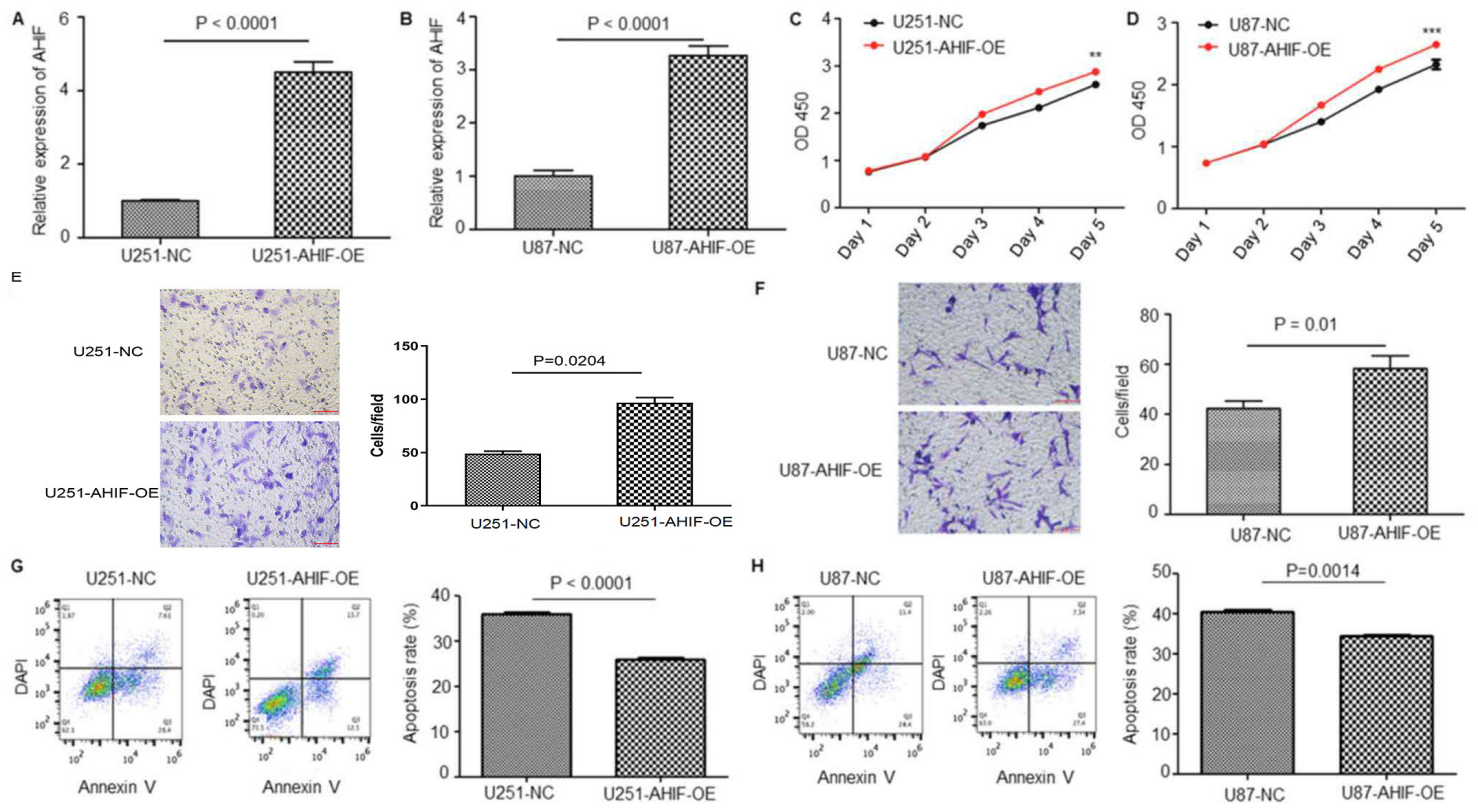
Overexpression of AHIF in GBM cells enhances viability, invasion and radioresistance. Relative AHIF levels in (A) U251-MG and (B) U87-MG cells with or without AHIF OE. Cell Counting Kit-8 analysis of viability in (C) U251-AHIF-OE and control cells, and (D) U87-AHIF-OE and control cells. Invasion analysis of (E) U251-AHIF-OE and control cells, and (F) U87-AHIF-OE and control cells. Apoptosis analysis of (G) U251-AHIF-OE and control cells, and (H) U87-AHIF-OE and control cells. All experiments were performed three times. AHIF, antisense transcript of hypoxia-inducible factor 1α; GBM, glioblastoma multiforme; OE, overexpression; NC, negative control; OD, optical density.

